# Analysis of road traffic injuries presented to the emergency department in the Eastern Province of Saudi Arabia: a hospital-based study

**DOI:** 10.25122/jml-2023-0316

**Published:** 2023-12

**Authors:** Amaar Amir, Baraa Amir, Asim Alghannam, Tareq Abdeen, Abdullah Al-Howaish, Rayan Alsheniber, Abdullah Al-Mulhim, Dunya Alfaraj

**Affiliations:** 1 College of Medicine, Imam Abdulrahman Bin Faisal University, Dammam, Saudi Arabia; 2 Department of Emergency Medicine, Imam Abdulrahman Bin Faisal University, Dammam, Saudi Arabia

**Keywords:** road traffic accidents, road traffic injuries, motor vehicle collision, accidents, time variation, CDC: Center for Disease Control, GNI: Gross National Income, KFUH: King Fahad University Hospital, MOH: Ministry of Health, RTA: Road Traffic Accident, RTI: Road Traffic Injury, SPSS: Statistical Package for Social Sciences, SICU: Surgical Intensive Care Unit, WHO: World Health Organization, IRB: Institutional Review Board

## Abstract

The increasing incidence of road traffic injuries (RTIs) has contributed to the disability and death of millions of people on both a national and global level. This retrospective study was conducted at King Fahad University Hospital (KFUH), Khobar, Saudi Arabia, and included all patients who presented at the emergency department due to road traffic accidents (RTAs) between January 1^st^, 2022, and December 31^st^, 2022. Patient data was retrieved from the health information system at KFUH. Descriptive and inferential analysis were performed with several variables analyzed using multivariate logistic regression and factorial ANOVA (MANOVA). During this period, 921 patients were treated at the hospital's emergency department. Of these, 611 (66.3%) were men and 310 (33.7%) were women. The most frequently affected age group was 16-25, representing 427 (46.4%) cases. Most patients were Saudi citizens (837, 90.9%). Among the patients, 19 (2.1%) required surgical treatment within 24 hours of the RTA, and 29 (3.1%) were admitted to the surgical intensive care unit (SICU). There were eight fatalities, representing 0.9% of the cases. January had the highest number of RTAs (12.7%). Moreover, 463 individuals (50.3%) had some form of injury, the most common type being lacerations and abrasions (n=228, 24.8%). Upper limb fractures were the most frequent type of fracture, occurring in 73 cases (7.9%). Being male (P=0.001), non-Saudi (P=0.014), and experiencing accidents during June and July (P=0.002) were associated with an increased prevalence of injury. Mortality had a statistically significant relationship with different patient age groups (P=0.014), patient citizenship (P=0.005), and length of hospital stay (P<0.001).

## INTRODUCTION

### Definitions

Road traffic accidents (RTA) are a major public health dilemma affecting countries all over the world [1]. Road traffic injuries (RTIs) refer to injuries resulting from the complex and nonlinear interaction between humans, vehicles, and the environment. RTAs result in a tremendous burden on the healthcare systems of varying countries, with some studies stating that RTAs are, in fact, the main cause of all trauma admissions to hospitals worldwide [2].

### Global epidemiology of RTIs

The World Health Organization (WHO) estimates that over a million people lose their lives due to RTAs on a yearly basis. Moreover, 20-50 million people worldwide experience non-fatal road traffic-related injuries, such as trauma and disabilities, requiring prolonged hospital stays. If efforts to combat RTAs are not improved or sustained, it is estimated that by 2030, RTAs will surpass all other causes of death globally [3]. In 2016, the WHO reported that the number of deaths secondary to RTAs reached an all-time high, with an estimated 1.35 million annual deaths. In comparison, the WHO reported in the year 2000 an estimated 1.15 million annual road traffic deaths. Countries in Africa and Southeast Asia had the highest rates of road traffic deaths, with 26.6 and 20.7 deaths per 100,000 individuals, respectively. Following these were the Eastern Mediterranean and Western Pacific, with rates of 18 and 16.9 deaths per 100,000 individuals, respectively. The regions with the lowest rate of road traffic deaths were America and Europe, with death rates of 15.6 and 9.3 per 100,000, respectively [4, 5]. It is reported that 90% of road traffic deaths occur in low and middle-income countries combined. It has been reported that approximately 73% of RTAs worldwide occur in young men below 25 years of age [6].

### Local epidemiology of RTIs in Saudi Arabia

RTIs represent a significant burden on a national scale, with a notable impact on Saudi Arabia. A recent study published in 2021 analyzed the data reported by the Red Crescent on RTIs in Saudi Arabia from 2016 to 2020. This study mentioned 63,737 road traffic accidents within 4 years and noted the highest incidence in the capital, Riyadh, at 13,656 cases (21.4%). Makkah was the second highest, reporting 9,673 cases, representing 15.2% of the incidents. The eastern province ranked third with 9,212 cases, 14.5% of the total. In contrast, the northern regions of Saudi Arabia, including Hail and Tabuk, reported lower numbers of road traffic accidents (2,438 [3.^8^%] and 1,947 [3.^1^%], respectively). Similarly, southern regions like Najran recorded fewer incidents, with 1,375 cases (2.2%), while other areas like Assir reported higher numbers at 7,864 (12.3%). Additionally, most RTIs involved male patients as opposed to female ones, with the former constituting 88.5% and the latter 11.5%. According to the Red Crescent, these numbers correlate well with most of the results of other studies conducted in the Kingdom [7-9].

### Time variation of RTIs

A study published in 2020 examined RTAs in the Eastern Province from 2009 to 2016, revealing that, on average, January and December had the highest incidence of RTAs within the calendar year, possibly due to the ‘tourism season’. This study also noted that September and October had the lowest incidence of accidents [10]. An article published in 2015 stated that the majority of accidents occur over the weekend, with the highest occurring on Thursday and the lowest occurring on Monday. Additionally, this study stated that the majority of incidents occurred during rush hour between 14:00 and 17:00 [11]. Furthermore, a study published in 2019, which utilized data from 2003 to 2013, identified a direct correlation between hazardous environmental conditions and the frequency of RTAs. In particular, dust and sandstorms were associated with an elevated incidence rate of accidents, which can be attributed to decreased visibility and increased traffic during such conditions [12].

### Classification of RTIs by location and type of injury

RTIs can be divided into several subtypes. A study conducted in 2019 categorized RTI injuries based on their nature, including lacerations, fractures, penetrating wounds, and more [3]. Another study, published in 2021, focused on orthopedic records and examined RTA patients admitted to the Armed Forces Hospitals in the Southern Region of Khamis Mushayt from 2011 to 2016 [13]. It was reported that the most common site of fractures were lower limbs (48.8%), with the least common being head and neck fractures (3.2%). Other percentages included upper limb fractures (28.1%), pelvic fractures (10.9%), spinal fractures (10%), and lastly rib fractures (8.1%) [13]. A study published in 2018 analyzed records of all RTI patients who underwent a pan-CT at King Fahad Military Medical Complex (KFMMC) from 2014 to 2017. In this retrospective study, injury patterns were sorted by region and were denoted head and neck (H), chest, abdomen, pelvis (C), or both (B). It was found that out of 305 RTA patients, 27 (8.8%) showed head and neck trauma (H), 93 (30.5%) had thoracic, abdominal, and pelvic trauma (C), while 98 patients (32.1%) suffered from both (B) injuries [14].

### Risk factors and prevention of RTIs

RTIs occur due to a complex interplay between the following three components: the environment, the vehicle, and human behavior. Environmental factors include visibility, road conditions, and weather. Factors related to vehicles include maintenance and engineering. Human factors include law violations and risky driving habits, such as excessive speeding, illegal turns, changing lanes without signaling, blocking intersections, driving through red lights, neglecting seatbelts [15], and overtaking other cars from the wrong side. The literature has also identified an association between drivers’ demographics and risky driving behavior, highlighting that young male drivers are more prone to engaging in behaviors that increase the risk of collisions [9]. Substantial efforts have been undertaken both domestically and on a global scale to address the underlying risk factors. The World Health Organization launched the Decade of Action for Road Safety campaign to combat RTIs, and the government of Saudi Arabia has approved the National Strategic Plan for Traffic Safety that aims to reduce the number of traffic injuries by 30% over 10 years following its enactment [9].

### Objectives

The primary objective of this study was to identify road traffic accidents (RTAs) and road traffic injuries (RTIs) among patients presenting to the emergency department of KFUH in 2022. The study aimed to examine the characteristics of these accidents, the types of injuries sustained, the demographic profiles of the patients, and their overall outcomes. Additionally, the study aimed to investigate the relationships between various independent variables and patient outcomes, making it an essential aspect of the current research.

## MATERIAL AND METHODS

### Study design and participants

This retrospective study was conducted at King Fahad University Hospital, Khobar, Saudi Arabia. The study relied on the institution's health information system to collect relevant data about the medical history of a specific group of patients. The study population consisted of individuals involved in RTAs who subsequently sought treatment at the emergency department of King Fahad University Hospital between 1 Jan 2022, and 31 Dec 2022. All patients falling within this timeframe and meeting the criteria for injuries sustained in in RTAs were included in the study without any restrictions based on age, gender, or nationality. For the purpose of this study, in RTAs were defined as any vehicular collision involving one party or more, and pedestrian collisions were also included.

### Study variables

Variables included demographic features such as patient age, gender, and nationality. Additionally, variables describing the incidents were considered, such as the month and time of day when the RTAs occurred. Furthermore, information surrounding the outcome was retrieved, including length of hospital stay, need for surgical intervention within 24 hours, admission to surgical intensive care unit, and mortality. Finally, details regarding the nature and location of injuries sustained were collected and included the presence or absence of upper limb fractures, lower limb fractures, skull and facial fractures, vertebral fractures, rib and sternal fractures, pelvic fractures, lacerations and abrasions, penetrating injuries, visible soft tissue injuries including bruising and swelling, amputations, thoracic visceral injuries (heart, great vessels, lungs, pleura, mediastinum, esophagus, and diaphragm), abdominal visceral injuries (stomach, liver, gallbladder, spleen, pancreas, kidney, small bowel, large bowel, mesentery, abdominal aorta and its branches, and inferior vena cava and its branches), pelvic visceral injuries (bladder, ureter, uterus, urethra, rectosigmoid, gonads), spinal injuries not including vertebral fractures, head or facial injuries not including fractures (intracranial hemorrhage, ocular injury, epistaxis, etc.), and loss of consciousness.

### Sampling and data collection

The research team retrieved data meeting the inclusion criteria from the KFHU health information system. Each researcher collected data from approximately 162 patients, resulting in 974 cases. Fifty-three cases were later omitted, likely due to being mislabeled in the health information system since the nature of the injuries was not a result of RTAs. The collected data was then categorized according to the mentioned variables and organized in an Excel spreadsheet. This organized data was used for subsequent analysis in the Statistical Package for Social Sciences (SPSS) program, version 28.

### Statistical analysis

The data analysis was conducted using SPSS version 28. Descriptive analysis was performed on all variables used in this study, with statistical significance set at <0.05, corresponding to a confidence interval of 95%. Descriptive results were presented as frequencies, percentages, medians, and interquartile ranges for non-parametric continuous variables. Multivariate logistical regression was performed to assess the relationship between the presence or absence of injury and the following variables: gender, nationality, age group, month, AM, or PM. Furthermore, the correlation between mortality and the variables mentioned above was studied via a similar test methodology. Length of stay and its correlation with the same variables were also analyzed via factorial ANOVA (MANOVA).

## RESULTS

**Figure 1:**
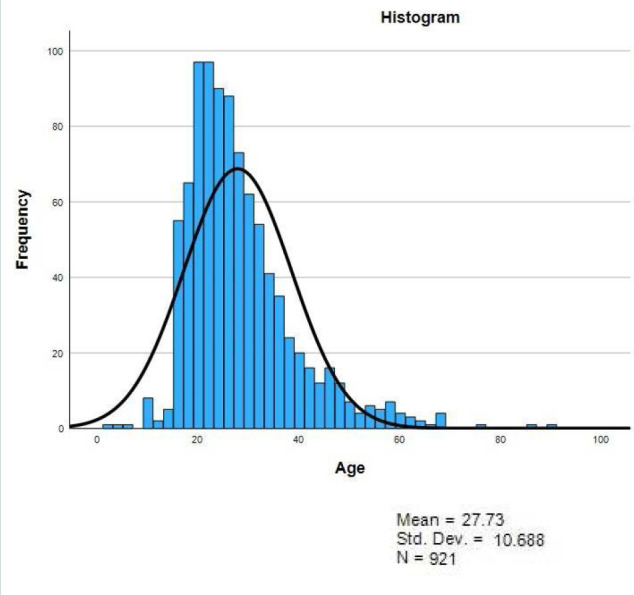
Histogram demonstrating the frequency of RTAs in different ages

**Figure 2:**
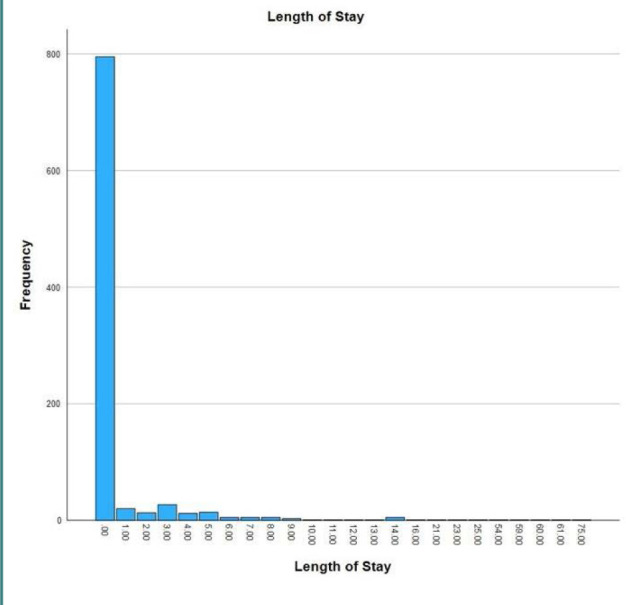
Length of stay among victims of RTAs

Our analysis included records from 921 patients treated for RTAs. The analysis showed that 611 (66.3%) were men, 427 (46.4%) were in the age group of 16-25 years, and 837 (90.9%) were Saudi citizens. The surgery-related characteristics showed that 19 (2.1%) patients were treated surgically within 24 hours of RTA, and 29 (3.1%) were admitted to the surgical intensive care unit (SICU). The mortality rate after admission was 0.9%. About 552 (59.2%) patients were admitted during the afternoon and evening hours. The months with the highest frequency of RTA cases were January (12.7%), May (12.3%), and February (11%) (Table 1). The median age and length of stay were 25 years with an interquartile range of 12 (Q1=20, Q3=32) and 0 with an interquartile range of 0 (Q1=0, Q3=0), respectively (Figures 1 and 2).

**Table 1:** Baseline characteristics of patients

		n	%
Gender	Male	611	66.3
	Female	310	33.7
Age group	<=15 years	38	4.1
	16 -25 years	427	46.4
	26 -35 years	294	31.9
	36 -45 years	100	10.9
	46 -65 years	54	5.9
	>65 years	8	.9
**Nationality**	Saudi	837	90.9
	Non-Saudi	84	9.1
Surgical intervention within 24hrs	Operated within 24hrs	19	2.1
	No operation within 24hrs	902	97.9
**SICU Admission**	Admitted to SICU	29	3.1
	Not admitted to SICU	892	96.9
**Mortality**	Expired	8	.9
	Recovered	913	99.1
Time of admission	AM	369	40.1
	PM	552	59.9
Month of admission	January	117	12.7
	February	101	11.0
	March	83	9.0
	April	83	9.0
	May	113	12.3
	June	72	7.8
	July	75	8.1
	August	56	6.1
	September	84	9.1
	October	42	4.6
	November	54	5.9
	December	41	4.5

**Figure 3:**
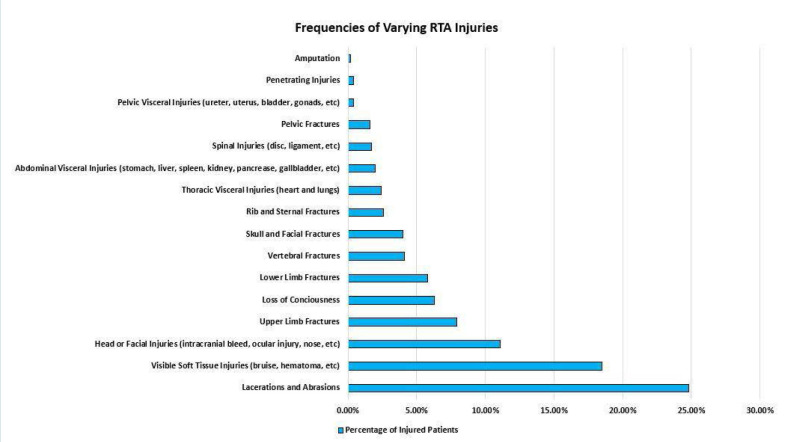
Frequency of various RTA injuries

The analysis showed that 463 (50.3%) had some form of injury. The frequencies of different types of injuries are depicted in Figure 3. The most common type of injury was lacerations and abrasions (24.8%), followed by visible soft tissue injuries (bruise, hematoma, etc.) (18.5%), head or facial injuries (11.1%), upper limb fractures (7.9%), and lower limb fractures (5.8%). The relationship between injury and other patient characteristics is shown in Table 2. Men had a significantly higher injury prevalence than women (54.2% vs. 42.6%), P=0.001. The prevalence of injury was significantly higher among non-Saudis compared to Saudis (63.1% vs 49%), P=0.014. Injuries secondary to RTAs during June and July had a significantly higher prevalence than in other months (P=0.002). Patients who had some kind of injury had significantly higher mortality rates compared to those who did not have any injury (P=0.005). Similarly, loss of consciousness was significantly higher among those who had some kind of injury (P<0.001). Multivariate logistic regression was conducted to assess the predictive factors for injury (Table 3). Male gender (OR=1.61, 1.21 - 2.15, P=0.001) was independently associated with injury prevalence.

**Table 2:** Relationship between prevalence of injury and other patient characteristics

			Presence of injury		Total	P value
			Present	Absent		
Age Group	≤ 15 years	n	24	14	38	0.097
		%	63.2%	36.8%	4.1%	
	16-25 years	n	201	226	427	
		%	47.1%	52.9%	46.4%	
	>26-35 years	n	153	141	294	
		%	52.0%	48.0%	31.9%	
	>36-45 years	n	46	54	100	
		%	46.0%	54.0%	10.9%	
	46-65 years	n	34	20	54	
		%	63.0%	37.0%	5.9%	
	≥ 66 years	n	5	3	8	
		%	62.5%	37.5%	0.9%	
Gender	Male	n	331	280	611	0.001
		%	54.2%	45.8%	66.3%	
	Female	n	132	178	310	
		%	42.6%	57.4%	33.7%	
Nationality	Saudi	n	410	427	837	0.014
		%	49.0%	51.0%	90.9%	
	Non-Saudi	n	53	31	84	
		%	63.1%	36.9%	9.1%	
Month	January	n	39	78	117	0.002
		%	33.3%	66.7%	12.7%	
	February	n	49	52	101	
		%	48.5%	51.5%	11.0%	
	March	n	38	45	83	
		%	45.8%	54.2%	9.0%	
	April	n	44	39	83	
		%	53.0%	47.0%	9.0%	
	May	n	56	57	113	
		%	49.6%	50.4%	12.3%	
	June	n	44	28	72	
		%	61.1%	38.9%	7.8%	
	July	n	51	24	75	
		%	68.0%	32.0%	8.1%	
	August	n	28	28	56	
		%	50.0%	50.0%	6.1%	
	September	n	43	41	84	
		%	51.2%	48.8%	9.1%	
	October	n	19	23	42	
		%	45.2%	54.8%	4.6%	
	November	n	28	26	54	
		%	51.9%	48.1%	5.9%	
	December	n	24	17	41	
		%	58.5%	41.5%	4.5%	
AM or PM	AM	n	194	175	369	0.253
		%	52.6%	47.4%	40.1%	
	PM	n	269	283	552	
		%	48.7%	51.3%	59.9%	
Mortality	Expired	n	8	0	8	0.005
		%	100.0%	0.0%	0.9%	
	Recovered	n	455	458	913	
		%	49.8%	50.2%	99.1%	
Length of hospital stay	0 days		347	452	799	0.002
			74.9%	98.7%	86.8%	
	1-2 days		29	4	33	
			6.3%	0.9%	3.6%	
	3-7 days		61	2	63	
			13.2%	0.4%	6.8%	
	8-14 days		17	0	17	
			3.7%	0.0%	1.8%	
	>=15 days		9	0	9	
			1.9%	0.0%	1.0%	
Loss of consciousness	Present	n	53	5	58	<0.001
		%	91.4%	8.6%	6.3%	
	Absent	n	410	453	863	
		%	47.5%	52.5%	93.7%	

**Table 3:** Logistic regression for predicting risk factors of injury

Dependent variable = Presence of injury		Odds ratio (95% CI)	P value
Independent variables	Age	0.89 (0.77 - 1.02)	0.089
	Gender = Male	1.61 (1.21- 2.15)	0.001
	Nationality	0.63 (0.39 -1.01)	0.055
	Month= January	3.18 (1.49- 6.79)	0.003
	Time of Admission	1.19 (0.90- 1.56)	0.215

The relationship between mortality and other patient characteristics is described in Table 4. It was found that mortality rates were significantly higher among the age group of ≥ 65 years (12.5%), 16-25 years (0.9%), and 26-35 years (1%) compared to other age groups (P=0.014). Non-Saudis had significantly higher mortality rates compared to Saudis (P=0.005). Patients with a loss of consciousness had significantly higher mortality rates than those who were conscious (P<0.001). Patients who stayed in hospitals for more than 15 days had significantly higher mortality rates compared to those who stayed fewer days (P<0.001). Other patient characteristics had no statistically significant association with mortality (P>0.05). Multivariate logistic regression was performed to assess the predictive factors for mortality (Table 5). Patients who had a spinal injury (OR=474.93, 6.74- 33458.97, P=0.005), head or facial injuries (OR=11.58, 1.00 -134.38, P=0.050), and loss of consciousness (OR=15.73, 1.15 -215.93, P=0.039) were independently associated with mortality.

**Table 4:** Relationship between mortality rate and other patient characteristics

			Mortality		Total	P value
			Expired	Recovered		
Age group	≤ 15 years	n	0	38	38	0.014
		%	0.0%	100.0%	4.1%	
	16-25 years	n	4	423	427	
		%	0.9%	99.1%	46.4%	
	>26-35 years	n	3	291	294	
		%	1.0%	99.0%	31.9%	
	>36-45 years	n	0	100	100	
		%	0.0%	100.0%	10.9%	
	46-65 years	n	0	54	54	
		%	0.0%	100.0%	5.9%	
	≥ 66 years	n	1	7	8	
		%	12.5%	87.5%	0.9%	
Gender	Male	n	7	604	611	0.203
		%	1.1%	98.9%	66.3%	
	Female	n	1	309	310	
		%	0.3%	99.7%	33.7%	
Nationality	Saudi	n	5	832	837	0.005
		%	0.6%	99.4%	90.9%	
	Non-Saudi	n	3	81	84	
		%	3.6%	96.4%	9.1%	
Month	January	n	2	115	117	0.623
		%	1.7%	98.3%	12.7%	
	February	n	0	101	101	
		%	0.0%	100.0%	11.0%	
	March	n	0	83	83	
		%	0.0%	100.0%	9.0%	
	April	n	0	83	83	
		%	0.0%	100.0%	9.0%	
	May	n	1	112	113	
		%	0.9%	99.1%	12.3%	
	June	n	1	71	72	
		%	1.4%	98.6%	7.8%	
	July	n	0	75	75	
		%	0.0%	100.0%	8.1%	
	August	n	0	56	56	
		%	0.0%	100.0%	6.1%	
	September	n	2	82	84	
		%	2.4%	97.6%	9.1%	
	October	n	1	41	42	
		%	2.4%	97.6%	4.6%	
	November	n	1	53	54	
		%	1.9%	98.1%	5.9%	
	December	n	0	41	41	
		%	0.0%	100.0%	4.5%	
Length of hospital stay	0 days	n	4	795	799	<0.001
		%	0.5%	99.5%	86.8%	
	1-2 days	n	0	33	33	
		%	0.0%	100.0%	3.6%	
	3-7 days	n	1	62	63	
		%	1.6%	98.4%	6.8%	
	8-14 days	n	1	16	17	
		%	5.9%	94.1%	1.8%	
	≥ 15 days	n	2	7	9	
		%	22.2%	77.8%	1.0%	
Loss of consciousness	Present	n	4	881	885	<0.001
		%	0.5%	99.5%	99.0%	
	Absent	n	2	7	9	
		%	22.2%	77.8%	1.0%	

**Table 5:** Logistic regression for predicting mortality risk factors after RTA

		Odds ratio (95% C.I.)	P value
Independent variables	Age	0.76 (0.23 -2.53)	0.657
	Gender	1.91 (0.05 - 72.24)	0.727
	Nationality	0.11 (0.01-1.20)	0.071
	Month	1.31 (0.79- 2.17)	0.293
	Time of admission	3.10 (0.28- 34.05)	0.355
	Surgery done in 24 hours	0.05 (0.00- 13.69)	0.290
	Admitted in SICU	10.56 (0.12 - 938.57)	0.303
	Upper limb fracture	3.07 (0.20- 47.17)	0.422
	Lower limb fracture	5.53 (0.24- 128.54)	0.286
	Skull facial fracture	5.35 (0.13 -224.68)	0.379
	Vertebral fracture	0.24 (0.00- 49.77)	0.601
	Rib sternal fracture	0.01 (0.00 -5.28)	0.148
	Pelvic fracture	4.13 (0.08- 220.08)	0.485
	Laceration abrasion	12.97 (0.73 -230.74)	0.081
	Penetrating injury	0.00 (0.00)	1.000
	Visible soft tissue injury	0.49 (0.03- 9.41)	0.636
	Amputation	0.00 0.00	1.000
	Thoracic visceral injuries	7.48 (0.10- 573.17)	0.363
	Abdominal visceral injuries	7.93 (0.29 -220.48)	0.222
	Pelvic visceral injuries	0.00 (0.00)	0.999
	Spinal injury	474.93 (6.74- 33458.97)	0.005
	Head or facial injuries	11.58 (1.00 -134.38)	0.049
	Loss of consciousness	15.73 (1.15 -215.93)	0.039

A factorial ANOVA (MANOVA) was conducted to examine the impact of various patient characteristics on the length of hospital stay following RTA. Several factors significantly influenced the duration of hospitalization, although the effect sizes were relatively small. The month of admission (July, October, December) (F(1,920)=4.895, ηp2=0.5%, P=0.027), admission to SICU (F(1,920)=121.163, ηp2=11.9%, P<0.001), lower limb fracture (F(1,920)=12.104, ηp2=1.3%, P=0.001), skull facial fracture (F(1,920)=9.10, ηp2=1.0%, P=0.003), vertebral fracture (F(1,920)=115.54, ηp2=11.4%, P<0.001), pelvic fracture (F(1,920)=10.318, ηp2=1.1%, P=0.001), thoracic visceral injuries (F(1,920)=4.03, ηp2=0.04%, P=0.045), pelvic visceral injuries (F(1,920)=9.667, ηp2=1.1%, P=0.002), spinal injury (F(1,920) =6.211, ηp2=0.07%, P=0.013), and head or facial injuries (F(1,920)=9,342, ηp2=0.1%, P=0.002), all demonstrated statistically significant associations with length of stay (Table 6).

**Table 6:** Factorial ANOVA model

Dependent Variable: Length of Stay (days)						
Source	Type III Sum of Squares	df	Mean Square	F	P value	Partial Eta Squared
Corrected Model	10847.716^a^	24	451.988	31.514	.000	.458
Intercept	607.463	1	607.463	42.354	.000	.045
Age	15.923	1	15.923	1.110	.292	.001
Gender	20.337	1	20.337	1.418	.234	.002
Nationality	6.548	1	6.548	.457	.499	.001
Month of admission	70.200	1	70.200	4.895	.027	.005
Time of admission	.314	1	.314	.022	.882	.000
Surgery done in 24 hours	7.720	1	7.720	.538	.463	.001
Admitted to SICU	1737.794	1	1737.794	121.163	.000	.119
Mortality	40.906	1	40.906	2.852	.092	.003
Upper limb fracture	25.838	1	25.838	1.801	.180	.002
Lower limb fracture	173.604	1	173.604	12.104	.001	.013
Skull facial fracture	130.517	1	130.517	9.100	.003	.010
Vertebral fracture	1657.153	1	1657.153	115.540	.000	.114
Rib sternal fracture	18.593	1	18.593	1.296	.255	.001
Pelvic fracture	147.985	1	147.985	10.318	.001	.011
Laceration abrasion	27.447	1	27.447	1.914	.167	.002
Penetrating injury	12.027	1	12.027	.839	.360	.001
Visible soft tissue injury	53.528	1	53.528	3.732	.054	.004
Amputation	.290	1	.290	.020	.887	.000
Thoracic visceral injuries	57.814	1	57.814	4.031	.045	.004
Abdominal visceral injuries	5.993	1	5.993	.418	.518	.000
Pelvic visceral injuries	138.656	1	138.656	9.667	.002	.011
Spinal injury	89.081	1	89.081	6.211	.013	.007
Head or facial injuries	133.987	1	133.987	9.342	.002	.010
Loss of consciousness	13.738	1	13.738	.958	.328	.001
Error	12851.029	896	14.343			
Total	24553.000	921				
Corrected Total	23698.745	920				

a. R Squared = .458 (Adjusted R Squared = .443)

## DISCUSSION

### Demographic characteristics

Demographic factors, specifically gender and age and their relationship with RTAs have consistently been a research topic. Analyzing the variations in gender and age groups frequently involved in RTAs can provide valuable insights that can assist local authorities in their efforts to educate and empower these populations. The ultimate goal is to reduce the incidence of vehicle collisions and the subsequent negative outcomes. The results of the current study indicate that men represented 66.3% of all RTA presenting to King Fahad Hospital of the University, while women represented a minority at 46.4%. While there was a statistically significant difference between men and women, this disparity was less pronounced compared to other regions within the Kingdom. For instance, a study in Riyadh showed a larger percentage of men involved in RTAs, representing 88% of all accidents [1]. Data obtained from Hail [3] reported 74.02% and 25.97% women, while the largest disparity between sexes was found in Najran at 92% male, with only 8% of cases involving women [8]. The variation in gender ratios could be attributed to several reasons, including differences in cultural norms across regions. The General Authority for Statistics of the Kingdom of Saudi Arabia issued a special report in 2020 showing that the Eastern Province is the second-highest region for issuing female driver licenses, representing approximately 27.9% [16]. This would be consistent with the narrow gender split of 19.9% in the current study in contrast to other regions displaying higher deltas.

Despite significant gender differences, the age groups commonly involved in RTAs in Saudi Arabia appear to be consistent across different regions. In our research, the most frequently affected age group comprised individuals between 16 and 25 years old, representing 46.4% of all RTA admissions to the University Hospital. The median age was 25, with an interquartile range of 12 (Q1=20, Q3=32). This aligns closely with findings from studies conducted in Riyadh, which reported a median age of 25 and an interquartile range of 14 (Q1=21, Q3=35) [1]. This higher prevalence of younger victims has been linked to riskier behavior. A study conducted in Oman investigating the age and gender association with RTAs concluded that the higher burden of RTIs was mainly attributed to the phenomenon of overspeeding, prevalent among those aged 20-29. The study also noted that the probability of severe injuries was highest among individuals aged 25-29, with an increased mortality rate observed between ages 20-24 [17]. Furthermore, the findings of our current research closely align with global averages in terms of gender and age disparities in RTAs. As reported by the WHO, 73% of all individuals involved in RTAs were young men, and they faced a threefold higher risk of fatal outcomes compared to young female drivers. RTIs were the leading cause of death for individuals between the ages of 5-29, according to the WHO, similar to the results obtained in our study [6].

### Time variation

Several studies [2, 11, 15] focusing on RTAs in the Kingdom of Saudi Arabia showed that an overwhelming majority occur in December and January, while the fewest accidents occur during the summer months. Jamal *et al*. [10] previously demonstrated that most RTAs in the Eastern Province of Saudi Arabia occurred in January and December, with a mean of 357 and 348, respectively. Conversely, the lowest number of accidents occurred during October and September, both with a mean of 297 cases. The current study showed similar results, with January reporting the highest number of documented RTAs (117 incidents), followed by May (113 incidents). Conversely, December and October had the lowest numbers, with 41 and 42 cases, respectively. One theory to explain the surge in RTAs during the winter months is the favorable climate and the increased availability of tourist attractions and events. Similarly, nighttime activities, where individuals can escape the scorching heat, tend to attract larger crowds than morning events. This could be one possible explanation for the patterns observed in our study, where the majority of documented RTAs occurred at night, accounting for 60% (552 cases), while the remaining 40% (369) took place in the morning hours. The increase in RTAs during May could be attributed to a combination of factors, such as the holy month of Ramadan and the start of exams for schools and universities where traffic congestion is at its peak [2].

### Nature and location of injuries

Alotaibi *et al*. [13] conducted a similar record-based case series in the Aseer region, specifically in Abha, between May 2011 and May 2016. Likewise, Ahmed *et al*. [3] conducted a more recent retrospective study in Ha'il, analyzing the records of 10,855 RTIs patients from January 2016 to December 2017. Both studies [13, 3] reported that lower limb fractures were the most observed injuries, followed by upper limb fractures. In contrast, our study showed a higher incidence of upper limb fractures compared to lower limb fractures. This decrease in lower limb fractures may be attributed to recent improvements in Saudi Arabia, in line with the Vision 2030 initiative, as these fractures typically result from high-impact incidents [18]. For example, Dahim [19] noticed a statistically significant decrease in RTAs, injuries, and fatalities following the increase in gasoline prices. Additional factors include the implementation of monitoring systems and strict traffic rules, increased public awareness, and improved overall road infrastructure. However, the impact of these factors is yet to be individually analyzed in the literature [19].

Additionally, our study identified common manifestations of RTAs, including lacerations and abrasions, bruises and hematomas, bone fractures, and head and facial injuries, with amputations being the least common form of injury reported. These findings align with the results of Ahmed *et al*. in Ha’il [3]. However, different regions reported varying findings. For instance, Alfalahi *et al*. [20] reported that the most common RTIs in Yemen were bruises and superficial wounds, followed by fractures, with internal organ injuries being the least common. Additionally, a study in Qassim concluded that the most common injuries involved the head and neck, followed by the lower limbs [21]. This variation in literature may be attributed to a multitude of regional factors that may influence the mechanism of injury, such as the average driver’s road experience, the nature of roads in urban versus rural areas [22], and the availability of speed cameras [23].

### Patient outcomes

According to the present study, 8 out of 921 patients experienced mortality, resulting in an overall mortality rate of 0.9%. This value is substantially higher than the national mortality rate of RTAs in Saudi Arabia reported for the year 2020 (0.18%), with 4618 deaths out of 25,561 reported RTIs [24]. These findings align with the 2016 WHO report, which indicated that Saudi Arabia experiences a higher number of road traffic accident deaths per 100,000 population (27.4) compared to most low-income countries. This disparity is even larger in other high-income countries such as the United States (10.6) and the United Kingdom (2.9) [2, 25]. Despite significant improvements in primary and secondary prevention measures, such as stricter seat belt legislation and widespread speed camera systems, positive changes in how RTAs are reported and documented may have contributed to the apparent increase in reported RTIs and their complications.

The current study has revealed a significant association between mortality and specific patient characteristics, including age, nationality, length of hospital stay, and the presence or absence of specific injuries. The mortality rate varied in different subpopulations of the study. For instance, individuals aged over 66 years had the highest mortality rate among all age groups (12.5%). In contrast, there were no recorded deaths among those aged less than 15 years or those between the ages of 36 and 65 years. This finding aligns with previous data from the Center for Disease Control and Prevention (CDC), with data suggesting that drivers older than 70 years old have a higher number of road traffic accident-related deaths compared to middle-aged drivers between 35 years old and 55 years old. This increased risk among elderly individuals is primarily due to their increased vulnerability to injury in the event of a crash event [26]. Furthermore, our study identified significant variations in mortality rates based on nationality, with non-Saudi individuals in our study experiencing a mortality rate six times higher (3.6%) than that of Saudi patients. One study reported that foreign travelers or tourists are at an increased risk of road traffic accident-related mortality, possibly due to their lack of familiarity with local roads and driving habits. Injuries are the leading cause of death among travelers, far exceeding deaths related to communicable infections, with RTAs accounting for 57% of deaths. However, pre-travel advice typically focuses on preventable diseases and obtaining necessary vaccines, often overlooking personal safety measures, including precautions against RTIs [27]. A survey revealed that 99% of travel clinics worldwide provide guidance on infection-related precautions, while only 70% discuss personal safety topics, including precautions against RTIs, among other concerns [28].

Additionally, our study identified specific types of injuries with an increased likelihood of causing death, such as spinal injuries, head and facial injuries, and loss of consciousness. Similarly, patients with longer hospital stays had significantly higher mortality rates than those with shorter stays. This data was consistent with other local studies conducted in the country [29]. Despite varying mortality rates associated with different population characteristics and injury patterns, road traffic-related mortality remains a significant issue affecting the entire Saudi Arabian population. Records from the Ministry of Health (MOH) hospitals indicate that 20% of hospital beds are occupied by victims of road traffic accidents, and 81% of in-hospital deaths are attributable to road traffic injuries [30]. Despite recent improvements and ongoing efforts in the country, with over 19 road traffic accident-related fatalities occurring daily [2], RTAs continue to represent a major health burden that needs to be further addressed.

### Preventive measures for RTAs

Given the significant societal and economic burden posed by RTAs, it is imperative to implement effective strategies to reduce their incidence. RTAs are preventable and can be tackled with a systemic approach to road safety. Firstly, local authorities must prioritize vehicle maintenance and ensure that vehicles are in optimal condition, adhering to safety standards. This includes properly functioning essential components such as lights, brakes, tires, mirrors, steering, and safety systems like sensors and automatic parking mechanisms. Secondly, road conditions need to be maintained frequently to prevent the prevalence of potholes and other defects that may induce accidents, and appropriate sidewalks should be created to allow pedestrians safe passage along the roads. Thirdly, obtaining a driver's license should involve rigorous examination and testing procedures to ensure drivers are well-qualified and knowledgeable about road safety. Lastly, a country should enact and enforce legislation to uphold strict driving standards. Penalties should be imposed for reckless driving and hazardous parking practices that could lead to RTAs.

### Limitations and recommendations

There are several possible limitations to be highlighted in this study. Firstly, this retrospective study discusses data from RTA patients presenting to KFHU. Therefore, the results do not represent the other regions of the Kingdom. Additionally, the population included in this study is 90.9% Saudi nationals and 9.1% non-Saudis, with the latter group being underrepresented, potentially introducing sampling bias. To address these limitations, we recommend using stratified random sampling to reduce bias. Additionally, future research should compare data from different regions of the Kingdom during the same time frame to improve the overall representativeness of findings for Saudi Arabia.

### Application

The findings of this study offer valuable insights that could aid local authorities in Al Khobar city by highlighting various demographic factors and the time variation of road traffic accidents in Al Khobar city. With this information, authorities could strategically reallocate resources during heightened RTA incidents to mitigate and decrease morbidity and mortality rates. Similarly, medical personnel should be better equipped to handle the most common injuries reported. Moreover, targeted awareness campaigns can be developed to raise awareness of RTAs, particularly among the demographic groups most frequently affected.

## CONCLUSION

In conclusion, the analysis of 921 patients treated for RTAs at King Fahad Hospital of the University revealed important insights into the demographic and clinical characteristics of this population. The majority of patients were male, aged between 16-25 years, and Saudi citizens. Lacerations and abrasions were the most common type of injury, and the prevalence of injury was higher in men and non-Saudi patients. Patients with spinal injury or head and facial injuries had a higher mortality rate compared to those who did not, and mortality rates were higher among patients aged >65 years and 16-25 years, non-Saudi patients, patients with a loss of consciousness, and those who stayed in hospitals for more than 15 days. Additionally, our study revealed variations in RTA frequency, particularly during the winter months. These findings have practical implications, as they can inform local authorities about the demographics and temporal patterns of RTAs. This knowledge can aid in designing targeted interventions and educational campaigns to reduce the incidence of vehicle collisions and their associated negative outcomes. Overall, the study provides valuable insights that can help develop targeted interventions to reduce the burden of RTAs in Saudi Arabia.

## Data Availability

Further data is available from the corresponding author upon reasonable request.
